# Electroactive Hydrogels Made with Polyvinyl Alcohol/Cellulose Nanocrystals

**DOI:** 10.3390/ma11091615

**Published:** 2018-09-04

**Authors:** Tippabattini Jayaramudu, Hyun-U Ko, Hyun Chan Kim, Jung Woong Kim, Ruth M. Muthoka, Jaehwan Kim

**Affiliations:** 1 Center for Nanocellulose Future Composites, Department of Mechanical Engineering, Inha University, 100 Inha-Ro, Nam-Gu, Incheon 22212, Korea; mr.jayaramudu@gmail.com (T.J.); lostmago@naver.com (H.-U.K.); Kim_HyunChan@naver.com (H.C.K.); jw6294@naver.com (J.W.K.); mwongelinruth@gmail.com (R.M.M.); 2 Laboratory of Material Sciences, Instituto de Quimica de Recursos Naturales, Universidad de Talca, Talca 747, Chile

**Keywords:** electroactive hydrogel, polyvinyl alcohol, cellulose nanocrystals, freeze–thaw method, actuation

## Abstract

This paper reports a nontoxic, soft and electroactive hydrogel made with polyvinyl alcohol (PVA) and cellulose nanocrystal (CNC). The CNC incorporating PVA-CNC hydrogels were prepared using a freeze–thaw technique with different CNC concentrations. Fourier transform infrared spectroscopy, thermogravimetric analysis, X-ray diffraction and scanning electron microscopy results proved the good miscibility of CNCs with PVA. The optical transparency, water uptake capacity and mechanical properties of the prepared hydrogels were investigated in this study. The CNC incorporating PVA-CNC hydrogels showed improved displacement output in the presence of an electric field and the displacement increased with an increase in the CNC concentration. The possible actuation mechanism was an electrostatic effect and the displacement improvement of the hydrogel associated with its enhanced dielectric properties and softness. Since the prepared PVA-CNC hydrogel is nontoxic and electroactive, it can be used for biomimetic soft robots, actively reconfigurable lenses and active drug-release applications.

## 1. Introduction

Hydrogels are hydrophilic three-dimensional network structures that are cross-linked physically or chemically and which maintain their structural integrity during formation [1]. They can hold large amounts of water molecules/biological solutions, which turn them into soft and viscoelastic materials. The soft, flexible, elastic and wet features of the hydrogels promote them as potential candidates for various biomedical and pharmaceutical applications including diapers, contact lenses, membranes, tissue engineering, drug delivery systems and biosensors [2,3,4,5]. Stimuli–response hydrogels change their structure (especially volume and shape) due to such conditions as pH, ionic strength, temperature and electric field [6,7]. Several studies revealed that acrylic acid and its polymers, as well as other hydrogels based on polymeric materials, are electric or pH responsive. However, acrylic acid is known to be toxic in nature [8]. This toxicity problem can be overcome by blending or reinforcing natural polymers into synthetic polymers. Accordingly, natural polymer-based hydrogels can show stimuli-responsive behavior, resulting in their high number of potential applications including biomimetic soft robots, haptic actuators, artificial muscles, active tunable lenses and active drug release. Thus far, many natural polymers have been used to develop hydrogels such as chitosan, cellulose, whey protein and carboxymethyl cellulose [9,10,11,12,13]. Among them, cellulose has merits in terms of renewability, biocompatibility, abundance, low price, superior mechanical properties and easy chemical modification.

Cellulose consists of crystal and amorphous parts connected in a row. Cellulose nanocrystal (CNC), a rod-like shaped nanocrystal, can be isolated from cellulose resources including wood pulp, tunicates, bacterial cellulose, cotton, ramie, hemp as well as other agricultural residues by treating them with acid hydrolysis [14,15,16]. CNC has a high degree of crystallinity, mechanical properties and a specific surface area [17,18]. The typical width of CNCs is in the range of 5–50 nm, but their length and width depend on the source and the process conditions. CNC produced by sulfuric acid hydrolysis is electrostatically stable and easily dispersed in polar aqueous suspensions due to the sulfate ester groups on their surfaces [19,20,21,22]. Based on their attractive characteristics, CNCs have been used as reinforcing agents for a wide range of applications in packaging films, nanocomposites, microchips, tissue engineering, actuators and sensors [23,24,25,26,27]. 

In hydrogels, reinforcement technology is playing a key role [21,26,27,28,29]. Cellulose can easily interact with various polar and water-soluble polymer materials. Thus, blending of CNC with hydrogels can reinforce the hydrogels in terms of mechanical properties and electromechanical properties. Especially the integration of CNC in hydrogels can increase their dielectric constant so as to improve its electroactive properties. With this background, this study aims to improve the transparency and electroactive properties of hydrogels by incorporating CNC into polyvinyl alcohol (PVA) to develop nontoxic electroactive hydrogels. PVA hydrogel was reported as an electroactive material [30] and showed higher transparency when the hydrogels were prepared using the solvent mixture of dimethyl sulfoxide (DMSO) and water (80:20 wt.%) [31]. PVA and CNC are known to be nontoxic. The basic physical properties of the prepared hydrogels including the swelling behavior, transparency and surface morphology were investigated using the water uptake capacity test, UV-vis spectroscopy and scanning electron microscopy (SEM). To study the CNC interaction and its structural and thermal characteristics, the prepared hydrogels were tested using Fourier transform infrared (FTIR) spectroscopy, X-ray diffraction (XRD) and thermogravimetric analysis (TGA). The mechanical properties of the prepared hydrogels were characterized using a universal testing machine. Furthermore, the actuation properties of the prepared hydrogels were tested by applying actuation voltage.

## 2. Materials and Methods

### 2.1. Materials

Cellulose cotton pulp (MVE, DPw-4580) of 98% purity was obtained from Buckeye Technology Inc. Poly (vinyl alcohol) (Mw = 85,000~124,000 g/mole, 99% hydrolyzed), sulfuric acid (H_2_SO_4_) and sodium hydroxide were purchased from Sigma-Aldrich Korea, Gyeonggi-do, South Korea. Dimethyl sulfoxide (DMSO) was purchased from Dae Jung chemicals & Metals Co. Ltd. (Gyeonggi-do, South Korea) Deionized (DI) water was used throughout the experiments. 

### 2.2. Preparation of CNC 

In this study, CNC was prepared using acid hydrolysis treatment. The preparation of CNC was described in Reference [19,20]; following is a brief explanation. The cotton pulp (20.0 g), a source of cellulose, was dispersed in H_2_SO_4_ (175 mL of 30% (*v*/*v*) aqueous) under mechanical stirring with 200 rpm and 6 h at 60 °C. An alkaline (NaOH, 1 M) pre-treatment was carried out on the cotton pulp to remove the non-cellulosic components and to prior obtain the high yield of CNC. The acid hydrolysis resulted in a suspension, and it was diluted (Ph = 7) by adding excessive deionized (DI) water, followed by centrifugation (11,000 rpm and 10 min). After this, the CNC suspension was homogenized and dialyzed overnight. A certain amount of homogenized CNC was dispersed in 20 mL of solvent mixture of DMSO and DI water (80:20 wt.%) by sonication for 1 h. Finally, 1% of CNC suspension was obtained and stored at room temperature until use. 

### 2.3. Preparation of PVA-CNC Hydrogels

For the preparation of the PVA-CNC hydrogels, 9 wt.% PVA solution and 1 wt.% CNC suspension were used. The transparent PVA solution was prepared by dissolving 9 g of PVA in a 91 g solvent mixture of DMSO and DI water (80:20 wt.%) by continuous stirring at 80 °C for 8 h under a nitrogen atmosphere. To the PVA solution, different amounts of 1 wt.% CNC suspension were added, while the weight of the PVA-CNC mixtures was kept constant at 20 g. After adding the CNC suspension, the PVA-CNC mixtures were sonicated for 20 min and then subjected to magnetic stirring for another 2 h (200 rpm) at 80 °C to obtain a homogeneous mixture. Finally, PVA-CNC hydrogels were obtained via a freeze–thaw process. The PVA-CNC mixtures were poured into a petri dish and subjected to three freeze–thaw cycles consisting of a 12 h freezing step at −20 °C, followed by a 6 h thawing step at room temperature. After finishing the three freeze and thaw steps, PVA-CNC hydrogels were formed. The prepared hydrogels were immersed in 100 mL of DI water in order to remove solvents and water-soluble/unreacted materials [32,33]. The DI water was changed every 8 h up to 3 days. The thickness of the prepared hydrogels was 4 ± 0.05 mm. The prepared PVA-CNC hydrogels were kept in DI water until use. The sample codes of the PVA-CNC hydrogels were designated as PVA-CNCx according to the amount of CNC suspension used in the hydrogels. Table 1 provides the feed composition ratio of PVA to CNC.

## 3. Characterization

### 3.1. Physical Properties

A water uptake capacity test of the prepared PVA-CNC hydrogels was carried out. The hydrogels were dried in an oven for 24 h at 60 °C until their weight reached saturation. The weight of the dried hydrogels were noted and immersed in a 100 mL beaker containing 50 mL distilled water at room temperature to equilibrate for up to 48 h. Then the samples were taken out and blotted with wiper paper to remove water on their surface and again reweighed using an analytical balance (GH-200, A&D weighing, Tokyo, Japan). The water uptake ratio, W.U., can be represented using the following equation:

W.U._(g/g)_ = (W_wet_ − W_dry_)/W_dry_, (1)

where W_wet_ and W_dry_ denote the weight of the equilibrated hydrogel at 48 h and initial weight of the dried hydrogel, respectively.

The optical transparency of the prepared PVA-CNC hydrogels was measured using a UV-visible spectrophotometer (HP8452A, Agilent, Santa Clara, CA, USA). For the measurement, the hydrogels were cut into the desired shape and the spectra range of 200–800 nm wavelengths were recorded.

A Scanning electron microscope (SEM, S-4000, Hitachi, Tokyo, Japan) was used to observe morphologies of the prepared hydrogels. To prepare specimens, the prepared hydrogels were freeze-dried and coated with platinum. The images were taken using the SEM, at 15 kV accelerating voltage.

### 3.2. FTIR, XRD and TGA

FTIR spectroscopy was used to study the transmission of light and the interaction of CNCs of the prepared PVA-CNC hydrogels. For the FTIR analysis, the samples were completely dried in a vacuum oven at 60 °C for 6 h. The FTIR spectra were recorded on a FTIR spectrometer (Bruker Optics, Billerica, MA, USA) with the range of 400–4000 cm^−1^ using the KBr disk pellet method and averaging 16 scans.

XRD patterns of the prepared CNC, PVA, and PVA-CNC hydrogels were recorded using an X-ray diffractometer (DMAX 2500, Rigaku, Japan), with Cu Kα radiation source (λ = 0.1542 nm) at 40 kV and 300 mA. The scan speed was 2° per min and the spectra of 2θ (Bragg angle) ranged from 2.5 to 60°. The thermal stability of the prepared CNC, PVA and PVA-CNC hydrogels was studied using a TGA (STA 409 PC, NETZSCH , Selb, Germanay) at a constant heating rate of 10 °C/min in the range of 30–600 °C under a constant nitrogen flow (20 mL/min).

### 3.3. Mechanical Testing

The compression test of the PVA-CNC hydrogels was conducted at a fully-hydrated stage and was followed by the ASTM D-882-97 test method using a universal test machine (Won Shaft Jeong Gong, Gyeonggi-do, South Korea) under the ambient condition with compression rate of 0.0005 mm/s. The size of the specimens was 20 × 20 × 5 mm^3^. The specimen was kept between two parallel plates and the upper plate pressed the specimen until it reached the maximum value. 

### 3.4. Actuation Test

**Figure 1:**
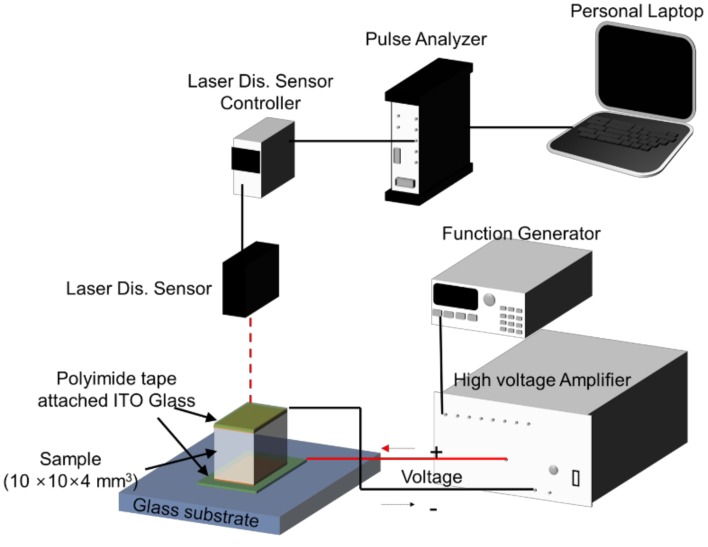
Schematic setup of the actuation test.

The actuation test was carried out using a laser displacement sensor (Keyance LK-G85, Tokyo, Japan), a high voltage amplifier (Model 10/10, Trek, Lockport, NY, USA) and a function generator (33220A, Agilent, Santa Clara, CA, USA). Figure 1 shows the schematic setup of the actuation test. Before conducting the actuation test, the hydrogel specimens (10 × 10 × 4 mm^3^) were equilibrated in DI water for 24 h and kept between two electrodes (polyimide tape attached to indium tin oxide coated glass (ITO glass)). A high voltage was applied on the electrodes via the function generator and the high voltage amplifier. The displacement of the hydrogel specimen was measured using the laser displacement sensor along with a data acquisition system (Pulse, B&K, Nærum, Denmark) connected to personal laptop. The actuation test was conducted at a constant environmental condition (25 °C, 95% RH) using an environmental chamber.

## 4. Results and Discussion

### 4.1. Physical Properties

**Figure 2:**
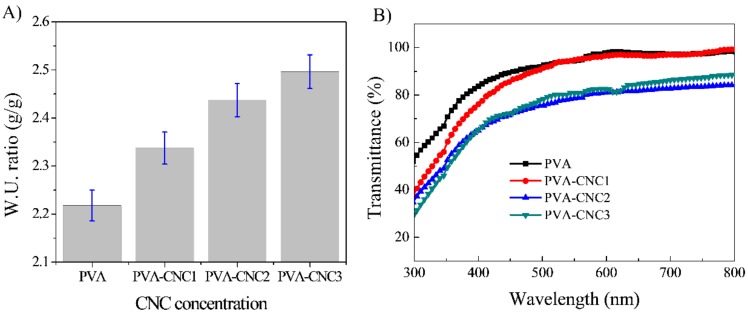
(**A**) Water uptake capacity of pure PVA and PVA-CNC hydrogels and (**B**) Optical transparency of the pure PVA and PVA-CNC hydrogels.

The water uptake ratios of the pure PVA and PVA-CNC hydrogels were calculated using Equation (1). Figure 2A shows the results for the pure PVA and the hydrogels. As the CNC concentration increased, the water uptake ratio consistently increased from 220% to 250%. This might be due to the fact that the hydrogen bonds between the CNC and PVA chains decreased the residual hydrogen bonds in the PVA chains, which resulted in increased water uptake [34].

The optical transparency of the prepared hydrogels was measured using the UV-vis spectroscopy at 300 to 700 nm. Figure 2B shows the optical transparency of the pure PVA and PVA-CNC hydrogels. The optical transparency taken at 500 nm of pure PVA was 92.4% and it decreased to 91.0, 77.7 and 75.9%, as the CNC concentration in the PVA-CNC hydrogels increased. Increasing the CNC concentration reduced the transparency due to CNC aggregation, which enhanced the turbidity of PVA hydrogel so as to decrease its transparency [20].

**Figure 3:**
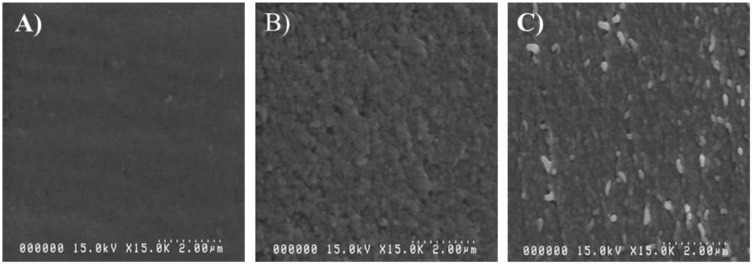
SEM images: (**A**) the pure PVA, (**B**) surface of PVA-CNC3 hydrogel and (**C**) cross-section of PVA-CNC3 hydrogel.

The SEM images of PVA-CNC3 hydrogel were taken to observe the morphology of the hydrogel. Figure 3A,B shows the surface morphologies of the pure PVA and PVA-CNC3 hydrogel, respectively. The pure PVA showed a smooth surface morphology, meanwhile the CNC-incorporated PVA-CNC3 hydrogel exhibited a uniform but rough surface. The CNCs were shown to be well-dispersed in the hydrogel. The cross-sectional SEM image of the PVA-CNC3 hydrogel (Figure 3C) showed that the rod-shaped CNCs were dispersed in the cross-sectional image of the hydrogel. This uniform dispersion might be associated with the interaction between the PVA and the CNC.

### 4.2. FTIR, XRD and TGA

**Figure 4:**
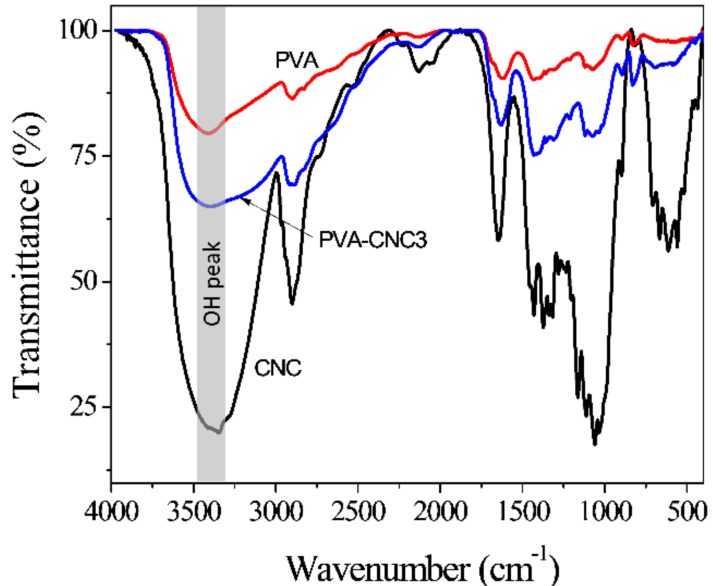
FTIR spectra of CNC, PVA, and PVA-CNC3 hydrogel.

To study the transmission of light and the interactions of CNC in the prepared PVA-CNC hydrogels, FTIR spectroscopy analysis was performed. Figure 4 shows the FTIR spectra of the prepared CNC, PVA, and PVA-CNC3 hydrogel. The O-H stretching vibration of the pure PVA is shown at 3422 cm^−1^. The characteristic peaks at 1070 cm^−1^ and 2901 cm^−1^ are related to stretching vibrations of the C-O and C-H. The peak at 1628 cm^−1^ is related to an acetyl group (C=O), which is induced from the preparation of PVA. A bending vibration related to CH_2_ groups is observed in the region of 1430–1446 cm^−1^ [10]. The FTIR spectra of the prepared CNC indicates the characteristic peaks assigned to cellulose I structures: Peaks are shown at 3374 cm^−1^ (O-H region), 2900 cm^−1^ (C-H stretching vibration), 1430 cm^−1^ (CH_2_ symmetric bending) and 1320 cm^−1^ (CH_2_ wagging at C-6). Peaks at 1065 cm^−1^, 1124 cm^−1^ and 1160 cm^−1^ demonstrate the presence of sulfate ester bonds, which are induced by the sulfuric acid hydrolysis for CNC preparation [35]. In the case of the PVA-CNC3 hydrogel, the O-H stretching vibration peak shifted to 3402 cm^−1^ due to the overlap of intermolecular hydrogen bonded O-H peaks from PVA-PVA and CNC-CNC. There is a new peak at 3201 cm^−1^ on the FTIR spectrum of PVA-CNC3. This peak might correspond to intermolecular hydrogen bonded between PVA and CNC. The result shows that CNC and PVA are well-interacted. This gives rise to the increase in the water uptake capacity of PVA-CNC hydrogels.

**Figure 5:**
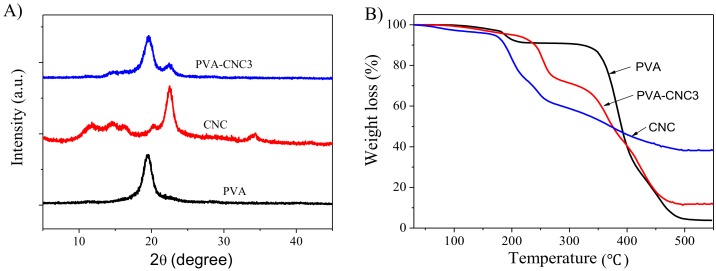
(**A**) X-ray diffraction patterns of CNC, PVA, and PVA-CNC3 hydrogel, (**B**) TGA curves of the pure PVA, CNC and PVA-CNC3 hydrogel.

Figure 5A represents XRD patterns that give crystalline information for the pure PVA, CNC and PVA-CNC3 hydrogel. PVA is known to be semi-crystalline in nature and the pure PVA shows the main strong diffraction intensity characteristic peak at 2θ = 19.5° [36] and the CNC sample exhibits four well-defined diffraction peaks at 2θ = 14.6, 16.2, 22.5, and 34.4°, which correspond to a typical cellulose I structure [22]. Note that the PVA-CNC3 hydrogel exhibits a similar diffraction peak at 2θ = 19.6°, with decreased intensity, and a shoulder peak at 2θ = 22.4°, with increased intensity, which suggests the physical interaction of PVA and CNC. This observation indicates that the incorporation of CNCs in PVA does not affect the crystalline structure of the PVA matrix. This means that the CNCs are well-dispersed in the PVA matrix so as to form the PVA-CNC hydrogel [37]. This fact was also confirmed for the FTIR spectra shown in Figure 4.

TGA measures the weight changes as a function of temperature. As the temperature increases, the weight of the sample decreases, indicating the continuous decomposition of the sample. Figure 5B shows the TGA curves of the pure PVA, CNC and PVA-CNC3 hydrogel. Below 150 °C, a minor weight loss occurred in all samples near 89 °C, which is associated with the evaporation of the absorbed water molecules. The pure PVA hydrogel showed mainly two weight-loss steps. The first weight loss started from 179 °C and finished at 216 °C (the weight loss was 8.9%), and was mainly associated with the dehydration of the hydroxyl groups by applying heat. The second weight loss started at 345 °C and degraded rapidly up to 500 °C (the weight loss was 95.4%), which was due to the degradation of the main chain. The PVA-CNC3 hydrogel showed two weight loss steps. The weight loss started from 212 °C and continually decreased up to 500 °C and a maximum 88.4% of weight loss was observed. Note that the PVA-CNC3 hydrogel showed higher thermal stability than the pure PVA, which might be due to the formation of the intermolecular bond between the CNC and the PVA. A similar observation has been reported previously [38,39]. In addition, the TGA spectrum of the CNC sample showed that the starting degradation temperature (160 °C) was lower than that of the pure PVA and the PVA-CNC3 hydrogel because CNC has many hydroxyl groups on its surface [40].

### 4.3. Mechanical Testing

**Figure 6:**
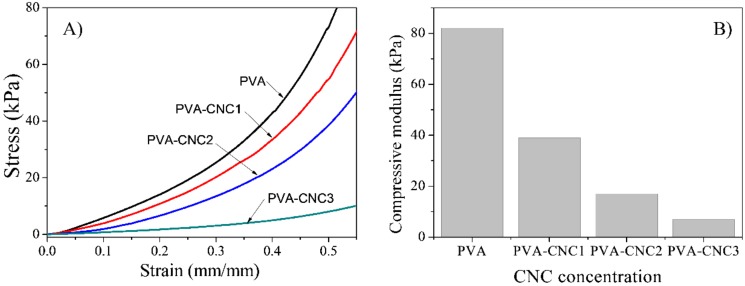
(**A**) Compressive stress–strain curves of PVA, PVA-CNC composite hydrogels and (**B**) compressive modulus.

The mechanical properties of the pure PVA and PVA-CNC hydrogels were studied using the universal testing machine. The test specimens were fully hydrated in distilled water. Figure 6A shows the compressional stress–strain curves of the pure PVA and PVA-CNC hydrogels with various CNC concentrations. Figure 6B shows the compressive modulus values of the hydrogels with different CNC concentrations. The modulus decreased with the increasing CNC concentration. The compressive modulus decrease of the PVA-CNC hydrogels can be surmised from the water uptake results. The mechanical property was inversely proportional to the water uptake capacity in the hydrogels: Increasing the water uptake capacity decreased the compressive modulus, due to the softening of the hydrogel structures. The CNC concentration plays an important role in the successful dispersion and formation of strong interfaces within the PVA polymer matrix. When the CNC concentration is above a critical value, the compressive strength of the hydrogels could be significantly decreased due to poor dispersion of the CNC as well as limited interfacial interaction between the CNC and PVA [41]. The compressive modulus of the pure PVA hydrogel was 82 kPa, and as the CNC concentration increased, it gradually decreased to 7 kPa for the PVA-CNC3 hydrogel. 

**Figure 7:**
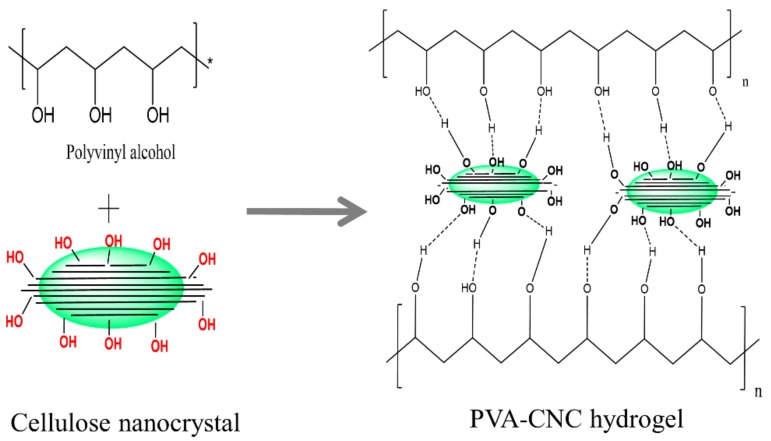
Formation of the PVA-CNC hydrogel.

Figure 7 shows the formation of PVA-CNC hydrogels. The hydroxyl groups of the PVA as well as on the surface of the CNC can interact with each other to form hydrogen bonds. However, as a large amount of CNC is dispersed in the hydrogel, for example PVA-CNC3, it seems that CNC aggregation occurs due to the hydrophilic nature of CNC, resulting in the evenly rough surface of the hydrogel.

### 4.4. Actuation Test

**Figure 8:**
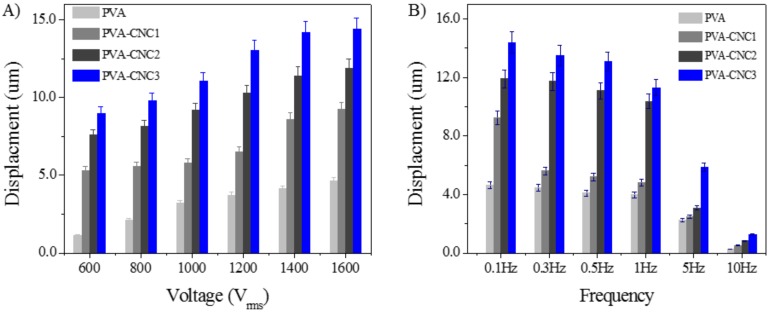
Actuation results for the pure PVA and PVA-CNC hydrogels: (**A**) actuation voltage variation at 0.1 Hz and (**B**) actuation frequency at 1.0 kV.

A soft actuator deforms in the presence of an external electric field. In this study, the deformation was defined as displacement and investigated in terms of the applied electric field and frequency. The actuation test of the prepared hydrogels was carried out in an aqueous swollen state in the DI water of the PVA-CNC hydrogels. Figure 8 shows the displacement of the hydrogels in terms of CNC concentration with the applied voltage and the frequency. Figure 8A shows the displacement of the hydrogels with a voltage change at 0.1 Hz. The displacement increased with increased voltage as well as increased CNC concentration. The maximum displacement of 14.4 µm was shown from the PVA-CNC3 hydrogel at 1.6 kV. This displacement corresponded to 3600 ppm strain and the applied voltage does corresponded to 0.4 V/µm electric field strength. This actuation performance is better than the cellulose hydrogel case (1800 ppm at 0.25 V/µm) [10]. Figure 8B displays the frequency-dependent displacement of the hydrogels under a constant voltage of 1.6 kV. The displacement output decreased with an increasing frequency. The CNC concentration played a significant role in the electroactive behavior of the PVA-CNC hydrogels, and the higher CNC concentration exhibited larger displacement than the pure PVA case. The PVA-CNC3 hydrogel showed higher displacement than the other hydrogels. This result is associated with the interfacial polarization between CNCs and the PVA polymer matrix [42]. It is a known fact that dispersed CNCs in a polymer matrix increases its dielectric properties, which is beneficial to improving its electroactive behavior.

Generally, an electroactive response occurs through a combination of piezoelectricity, electrostriction, flexoelectricity, electrostatic effect (Coulombic force), electrophoretic effect, electrochemical effect (ion migration) and electroosmotic interaction [5]. In the PVA/DMSO hydrogel, electrostrictive interaction was claimed as the mechanism causing its electroactive response since the displacement was proportional to the square of the electrical actuation voltage [30]. In some electroactive materials, it is hard to clarify the mechanism because although the same materials are used, different morphologies or physical/chemical properties could result in different mechanisms of electroactive behavior.

The prepared PVA-CNC hydrogel is not an ionic hydrogel. Although a sulfate ester bond peak was observed from the FTIR, this peak was caused by the sulfuric acid hydrolysis for the CNC preparation. During the CNC preparation, sulfuric acid was applied to isolate CNC from the pulp and some of the sulfuric ions remained on the surface of the CNCs. However, the remnant sulfuric ions were not significant because CNCs were dialyzed and washed with DI water several times. Thus, we believe that the prepared hydrogel was a nonionic hydrogel and the ion migration effect was not significant in the prepared hydrogel.

When the displacement and voltage curves are considered, as shown in Figure 8A, the displacements increased linearly or quadratically with the actuation voltage, as well as CNC concentration. This may give piezoelectricity in the hydrogel. However, it is very hard to possess dipole domains in the hydrogel. Thus, the prepared hydrogel was far from a piezoelectric material. On the other hand, as increasing the CNC concentration causes the compressive modulus to decrease, which is beneficial in terms of increasing the electrostatic effect associated with the Coulombic force. Under Coulombic force, a large strain can be produced when the electroactive material is soft. This is a well-known fact for dielectric elastomer electroactive polymers. In summary, since the PVA-CNC hydrogel is a soft, nonionic hydrogel, and it has high dielectric properties, the electrostatic effect may be its dominant actuation mechanism.

## 5. Conclusions

In this research, nontoxic, soft and electroactive PVA-CNC hydrogels were prepared using the freeze–thaw process with different CNC concentrations, and their characteristics were analyzed. The water uptake capacity of the hydrogels increased and the compressive modulus decreased as the CNC concentration increased. The optical transparency of the prepared hydrogels was inversely proportional to the CNC concentration. The thermogravimetric analysis and scanning electron microscopy results showed good miscibility of CNC with PVA. The CNC incorporated PVA-CNC hydrogels showed an improved displacement output in the presence of the electric field and with the increasing CNC concentration. The maximum 3600 ppm strain was produced under 0.4 V/µm electric field strength from the PVA-CNC3 hydrogel. The displacement improvement of the PVA-CNC hydrogels is associated with their enhanced dielectric properties and reduced compressive modulus. Since the PVA-CNC hydrogel is nonionic, soft, and it possible has strong dielectric properties, the electrostatic effect may be its dominant actuation mechanism. Since the prepared PVA-CNC hydrogel is nontoxic and electroactive, it may be a promising material for biomimetic soft robots, actively reconfigurable lenses and active drug-release.

## Figures and Tables

**Table 1:** Feed composition ratio of PVA-CNC hydrogels.

Hydrogels	Weight of 9 wt.% PVA (g)	Weight of 1% wt. CNCs (g)
PVA	20	0
PVA-CNC1	17	3
PVA-CNC2	15	5
PVA-CNC3	13	7
